# Supplementary figures and images for: The expression of essential selenoproteins during development requires SECIS-binding protein 2–like
Source: Life Sci Alliance. 2022 Feb 24;5(5):e202101291. doi: 10.26508/lsa.202101291 (PMC8881744; doi:10.26508/lsa.202101291)

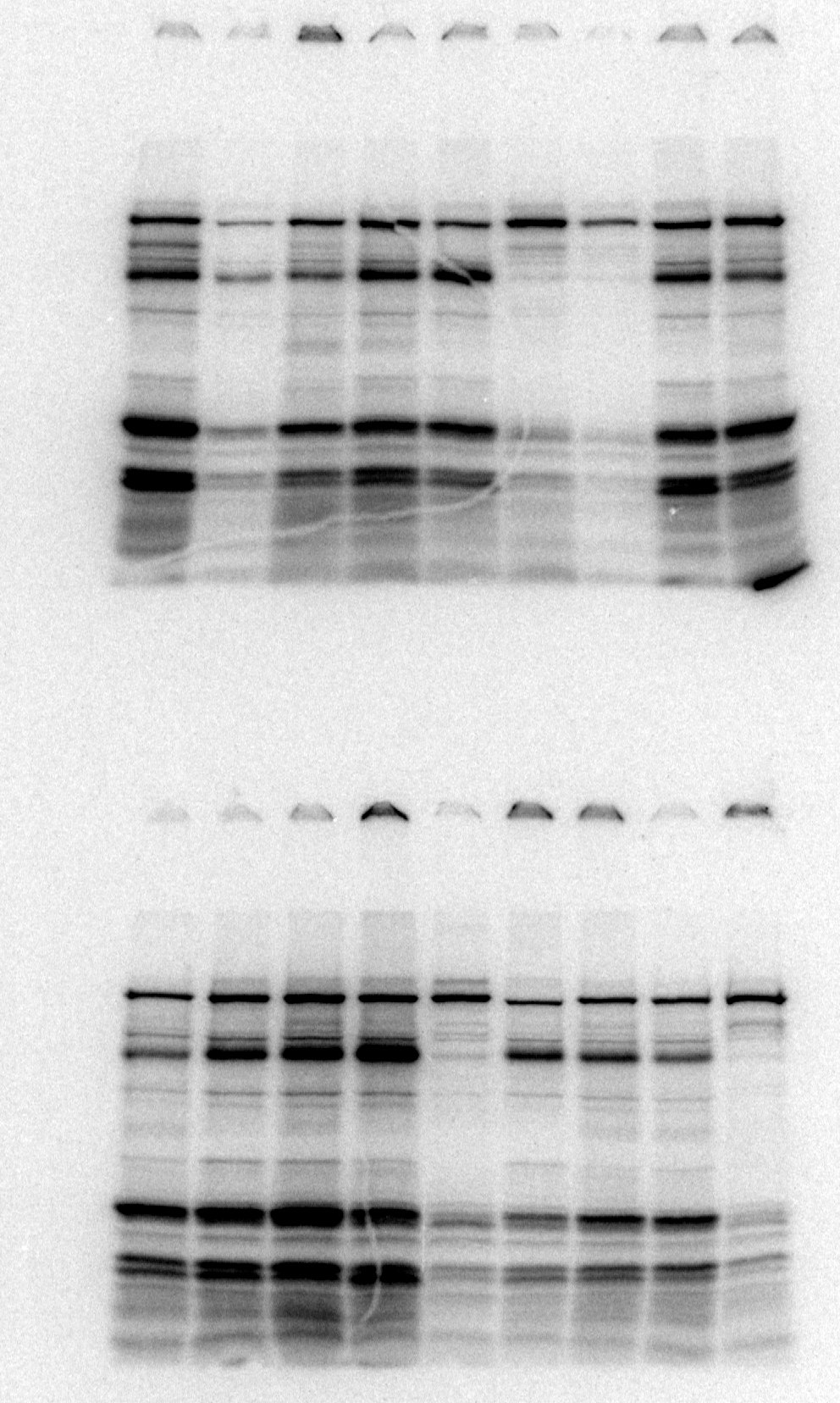

Supplement: Supplementary file 1 [file LSA-2021-01291_SdataF5.jpg]
